# Supplementary material for: TRIM28 modulates nuclear receptor signaling to regulate uterine function
Source: Nat Commun. 2023 Aug 1;14:4605. doi: 10.1038/s41467-023-40395-7 (PMC10393996; doi:10.1038/s41467-023-40395-7)
Supplement: Supplementary file 1 — Supplementary Figure [file 41467_2023_40395_MOESM1_ESM.pdf]

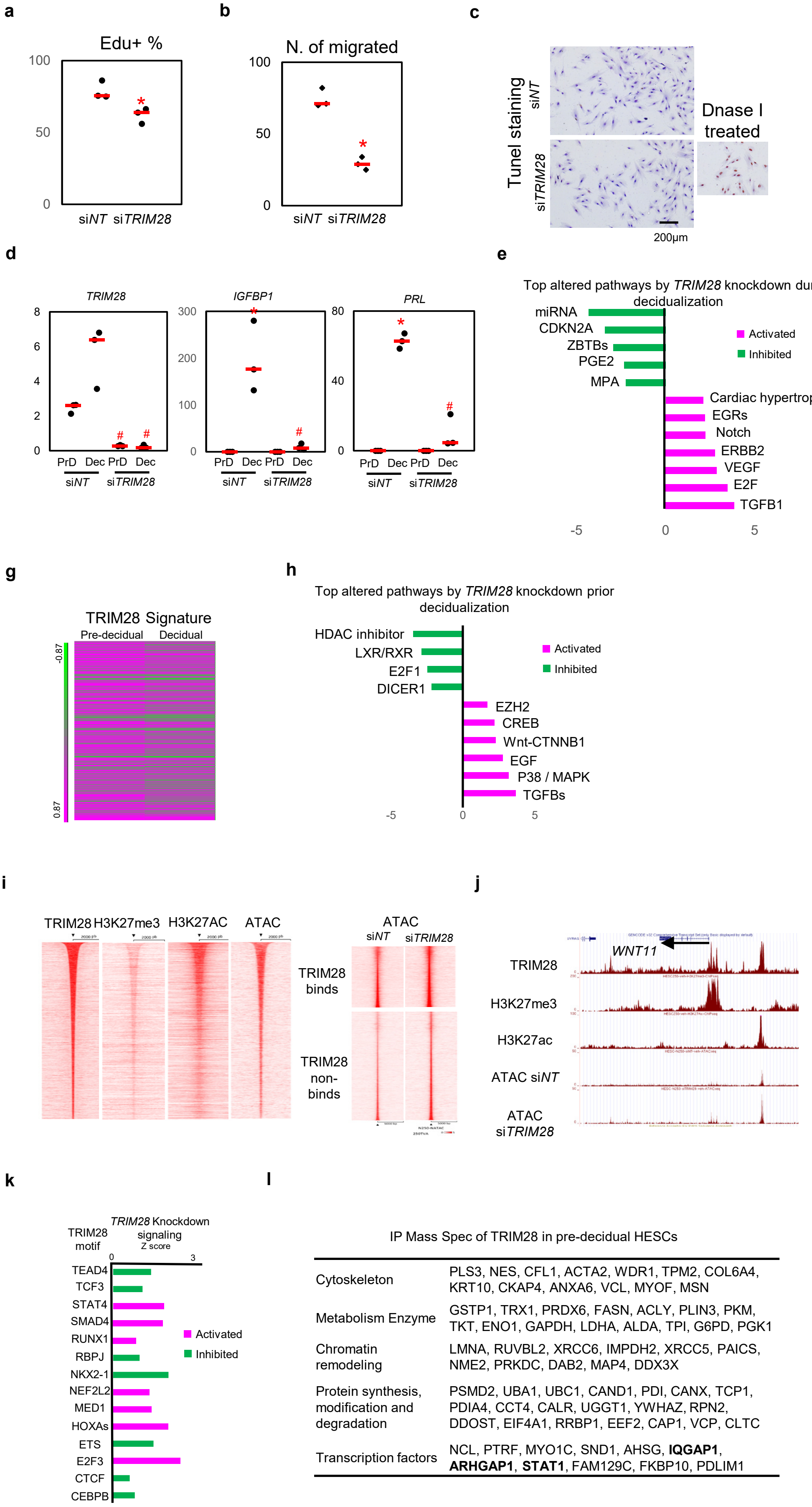

Cytoskeleton	PLS3, NES, CFL1, ACTA2, WDR1, TPM2, COL6A4, KRT10, CKAP4, ANXA6, VCL, MYOF, MSN
Metabolism Enzyme	GSTP1, TRX1, PRDX6, FASN, ACLY, PLIN3, PKM, TKT, ENO1, GAPDH, LDHA, ALDA, TPI, G6PD, PGK1
Chromatin remodeling	LMNA, RUVBL2, XRCC6, IMPDH2, XRCC5, PAICS, NME2, PRKDC, DAB2, MAP4, DDX3X
Protein synthesis, modification and degradation	PSMD2, UBA1, UBC1, CAND1, PDI, CANX, TCP1, PDIA4, CCT4, CALR, UGGT1, YWHAZ, RPN2, DDOST, EIF4A1, RRBP1, EEF2, CAP1, VCP, CLTC
Transcription factors	NCL, PTRF, MYO1C, SND1, AHSG, **IQGAP1**, **ARHGAP1**, **STAT1**, FAM129C, FKBP10, PDLIM1

Supplementary Fig.1. *TRIM28* knockdown impaired the biology of decidual and pre-decidual HESCs. Percentage of Edu positive (**a**), number of migrated (**b**) and Tunel staining (**c**) of HESCs. N=100 cells examined over three independent experiments and repeated in three primary cell lines with similar results. Two-sided student's t test. *p<0.05. **d**. Real-time PCR of *TRIM28*, *IGFBP1*, *PRL* in pre-decidual and decidual HESCs. N=200,000 cell examined over three independent experiments. One-way ANOVA with post-hoc turkey's test. *p<0.05, compared to PrD. #p<0.05, compared to siNT. **e**. Top altered pathways after si*TRIM28* treatment during decidualization. Green means inhibition. Magenta means activation. **f**. Genome browser of PR and TRIM28 co-binding peaks at *PTGFR*, *IL6*, *FZD7* and *F2RL1* in decidual HESCs. **g**. Heatmap of TRIM28 gene signature in pre-decidual and decidual HESCs. **h**. Top altered pathways in pre-decidual HESCs after *TRIM28* knockdown. Heatmap of chromatin accessibility (**i**) and genome browser (**j**) of TRIM28, H3K27AC, H3K27me3, and ATAC-seq in the pre-decidual HESCs. **k**. Enriched motif in TRIM28 binding peaks and the associated pathway activity after TRIM28 Knockdown. Z score is the pathway activity. Green is inhibition. Magenta is activation. **l**. Top TRIM28 partners in pre-decidual HESCs by IP-mass spec. PrD: Pre-decidual; Dec: Decidual.

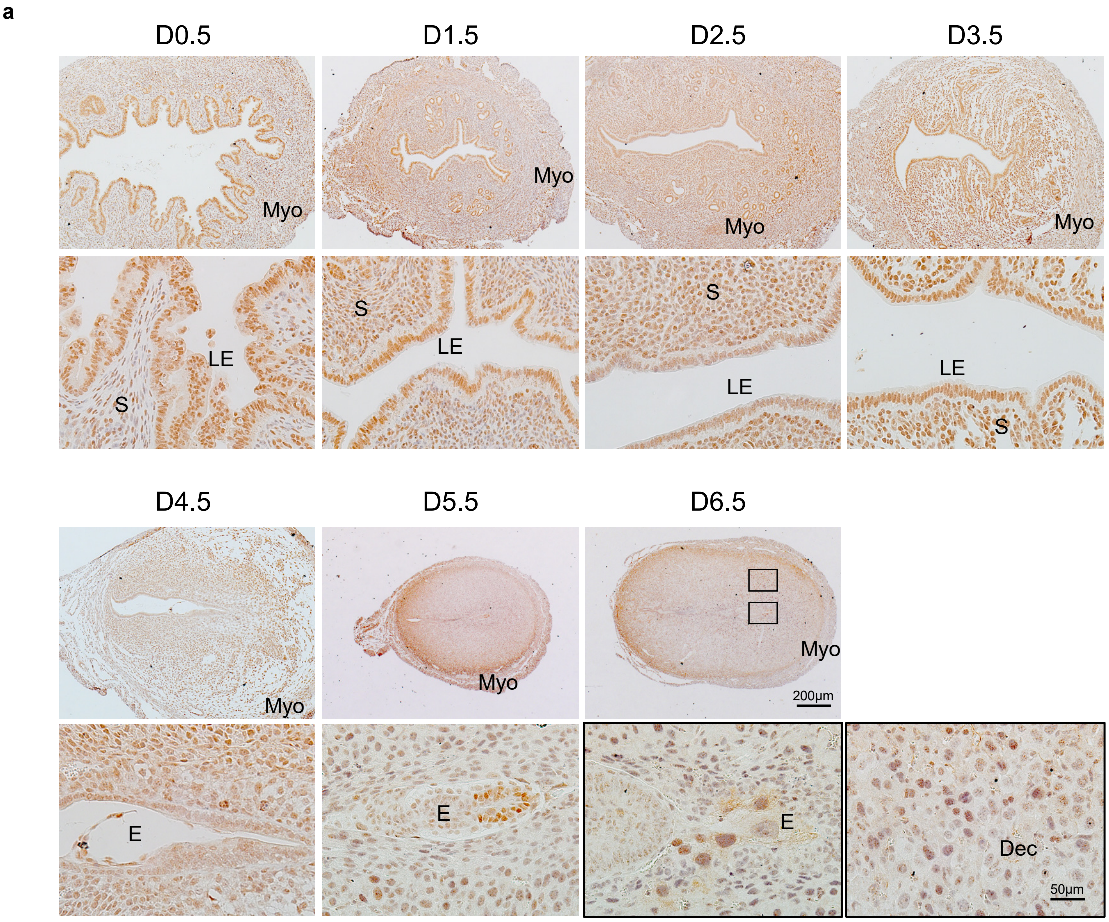

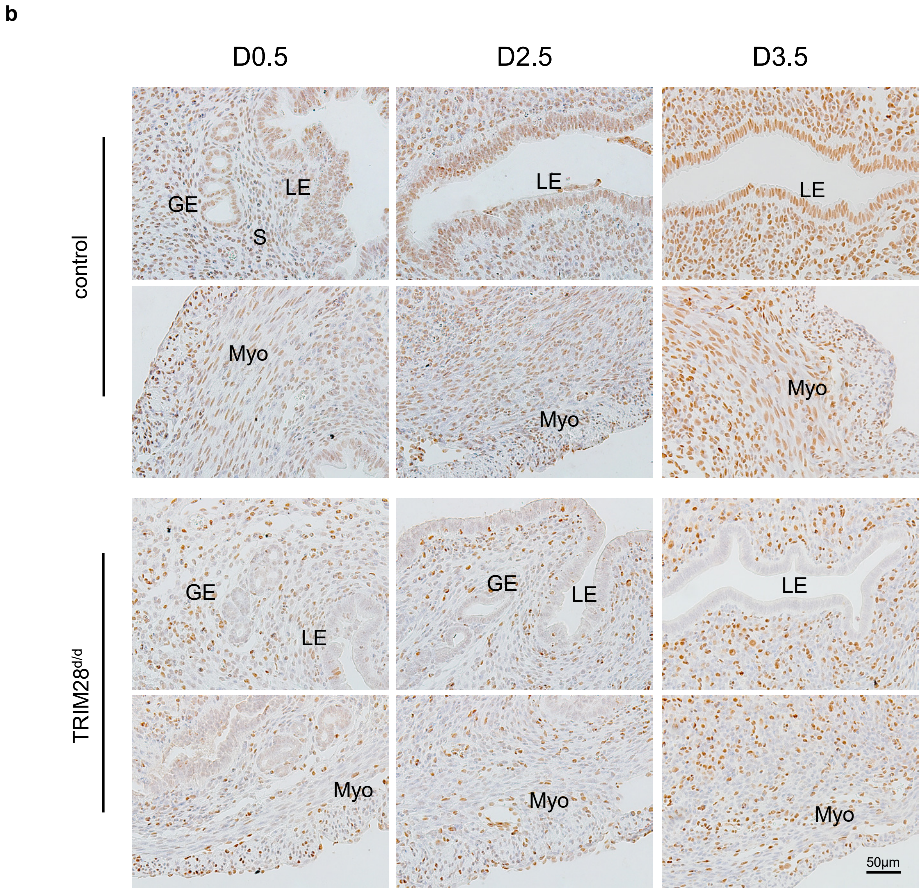

Supplementary Fig 2. TRIM28 expression in the $TRIM28^{f/f}$ and $TRIM28^{d/d}$ mice during early pregnancy. a. Immunohistochemistry (IHC) of TRIM28 in the mouse uterus at D0.5-7.5. **b**. Immunohistochemistry of TRIM28 in $TRIM28^{f/f}$ and $TRIM28^{d/d}$ mouse uterus at D0.5-3.5. E: Embryo; GE: Glandular epithelium; LE: Luminal epithelium; S: Stroma; Myo: Myometrium. The IHC was repeated in three different mice per group with similar results.

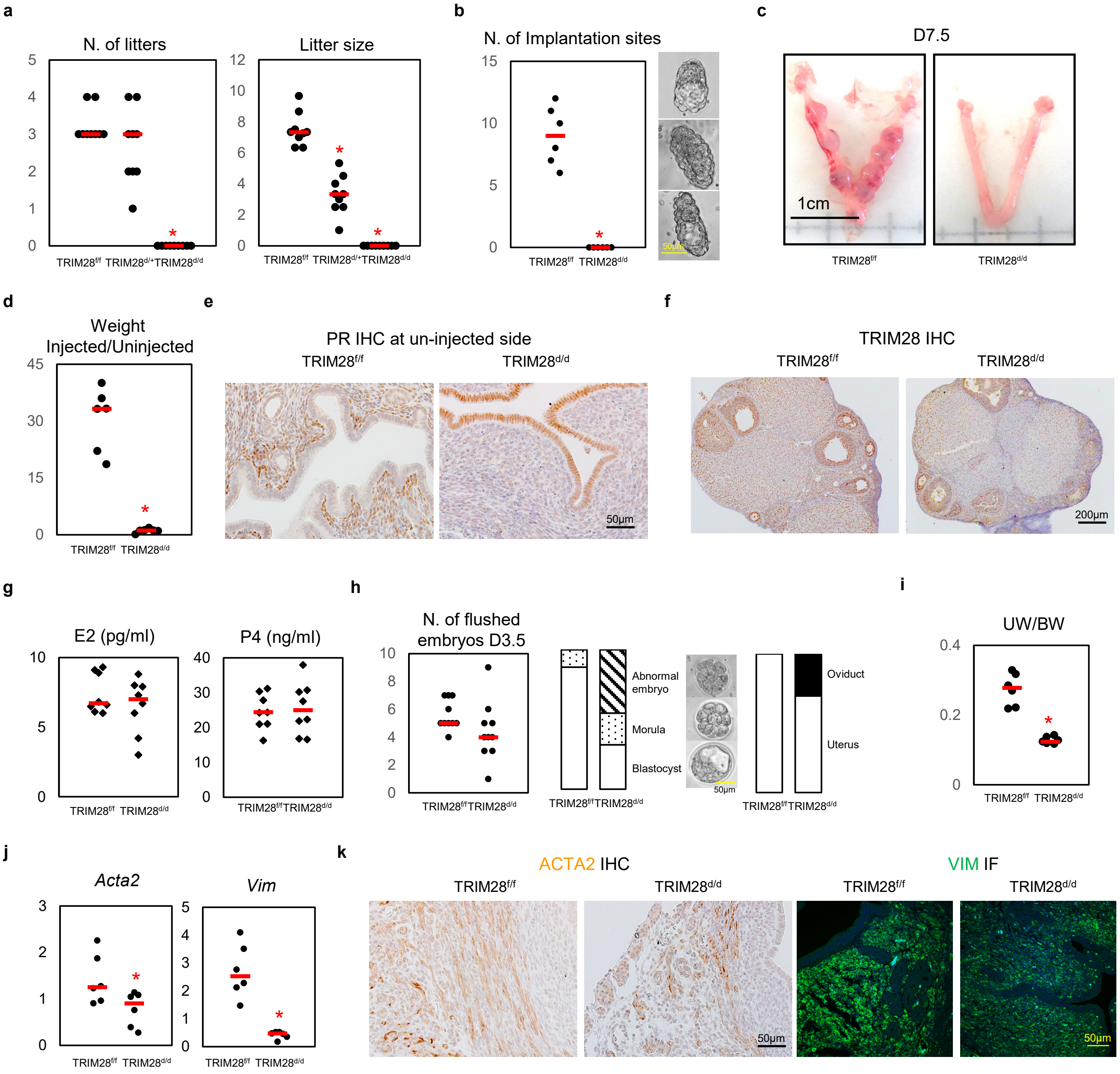

Supplementary Fig. 3. TRIM28$^{d/d}$ mice were infertile. **a**. The number of litter and litter size in the TRIM28$^{f/f}$, TRIM28$^{d/+}$ and TRIM28$^{d/d}$ mice after 6-month breeding trial. N=10 biologically independent samples per group. **b**. The number of embryo implantation sites in the TRIM28$^{f/f}$ and TRIM28$^{d/d}$ mice at D4.5. Blastocysts with normal morphology were flushed out from the TRIM28$^{d/d}$ mice. N=6 biologically independent samples. **c**. The representative uterine pictures at D7.5. **d**. The weight ratio of oil injected over un-injected side uterus within the same mouse upon artificial decidualization. N=6 biologically independent samples per group. **e**. Immunohistochemistry of PR at un-injected side uterus. **f**. Immunohistochemistry of TRIM28 in mouse ovaries. **g**. Serum levels of 17β-estradiol (E2) and progesterone (P4). N=8 biologically independent samples per group. **h**. The number of flushed embryos from the D3.5 mouse uterus and oviduct. The percentage of blastocyst, morula and bad embryos from the flushed embryos. N=10 biologically independent samples per group. The percentage of embryos flushed out from the uterus or oviduct. **i**. The ratio of uterine weight over body weight. N=6 biologically independent samples per group. **j**. The real-time PCR of *Acta2* and *Vim* of the TRIM28$^{f/f}$ and TRIM28$^{d/d}$ mice at 3.5. N=6 biologically independent samples per group. **k**. The immunohistochemistry (IHC) of ACTA2 and immunofluorescence (IF) of VIM. Two-sided student's t test. *p<0.05. The IHC and IF were repeated in three different mice per group with similar results.

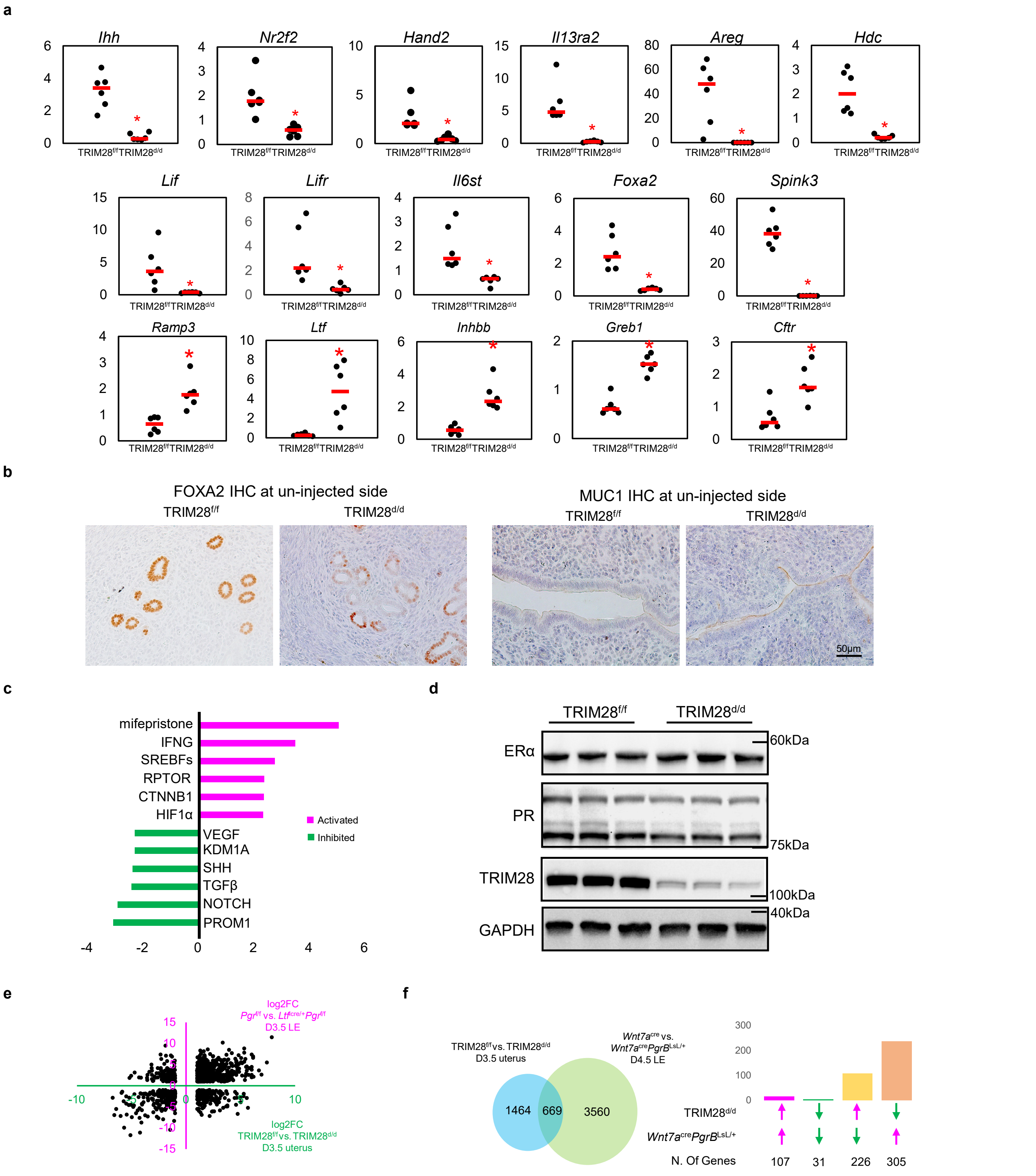

Supplementary Fig. 4. TRIM28 altered molecular signaling in D3.5 mouse uterus. **a**. Realtime PCR of PR target genes: *Ihh*, *Nr2f2*, *Hand2*, *Il13ra2*, *Areg*, *Hdc*; gland specific genes *Lif*, *Lifr*, *Il6st*, *Foxa2*, *Spink3* and estrogen specific genes *Ramp3*, *Ltf*, *Inhbb*, *Greb1*, *Cftr*. N=6 biologically independent samples per group. **b**. Immunohistochemistry of FOXA2 and MUC1. **c**. Top altered pathways in TRIM28$^{d/d}$ uterine transcriptome. **d**. Western blot of ERα, PR, TRIM28 and GAPDH in the mouse uterus. The western blot were performed in three different mice per group with similar results. **e**. The correlations of DEG fold changes between TRIM28$^{d/d}$ and epithelial PR knockout (*Ltf*$^{icre/+}$*Pgr*$^{f/f}$). **f**. The overlapped DEGs between TRIM28$^{d/d}$ and epithelial PRB overexpressed (*Wnt7a*^{cre}*PgrB*$^{LsL/+}$) uterus. Two-sided student's t test. *p<0.05.

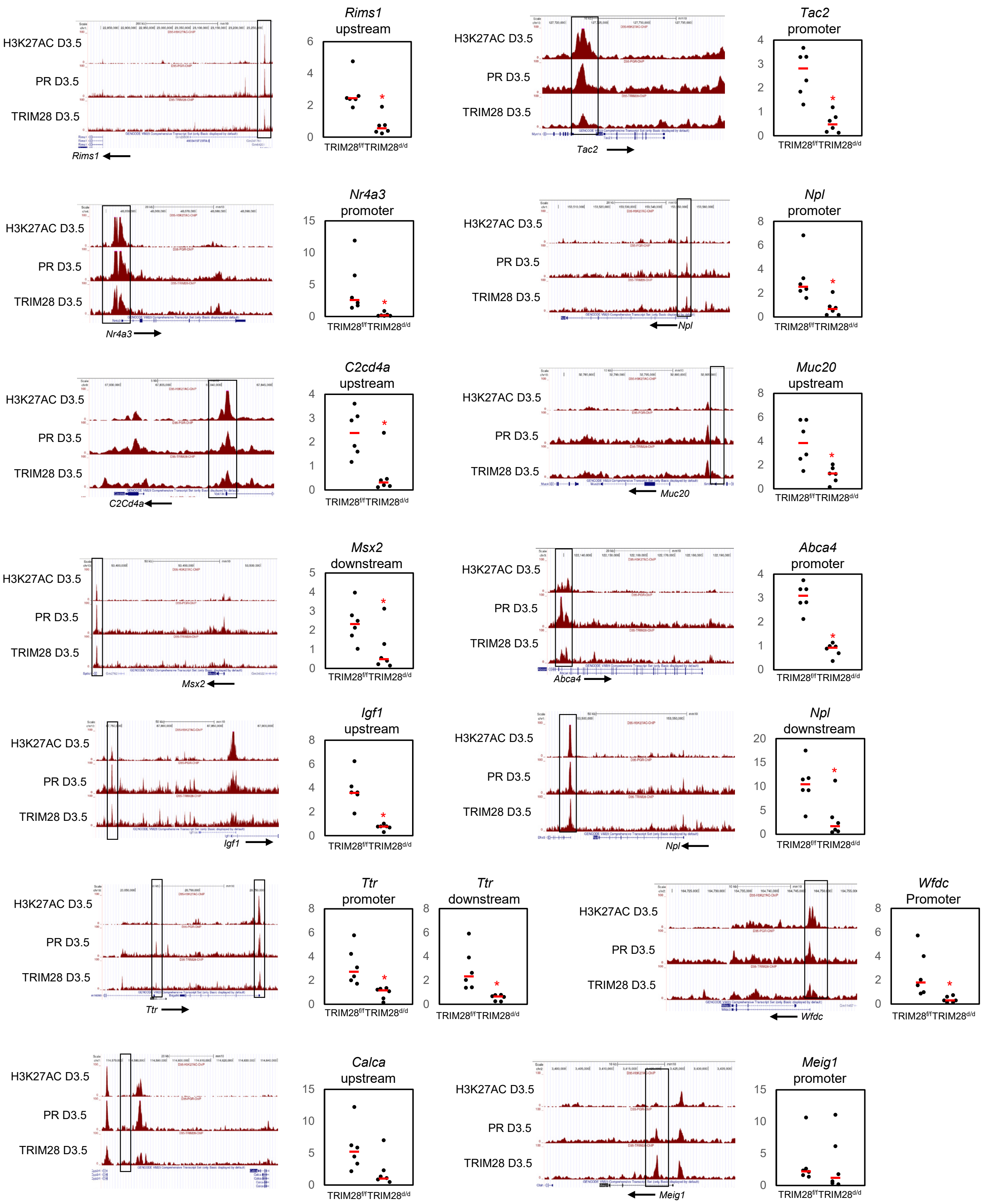

Supplementary Fig. 5. The genome browser of PR, TRIM28, H3K27AC ChIP-Seq, and PR ChIP-qPCR in D3.5 TRIM28$^{f/f}$ and TRIM28$^{d/d}$ uterus. N=6 biologically independent samples per group. Two-sided student's t test. *p<0.05.

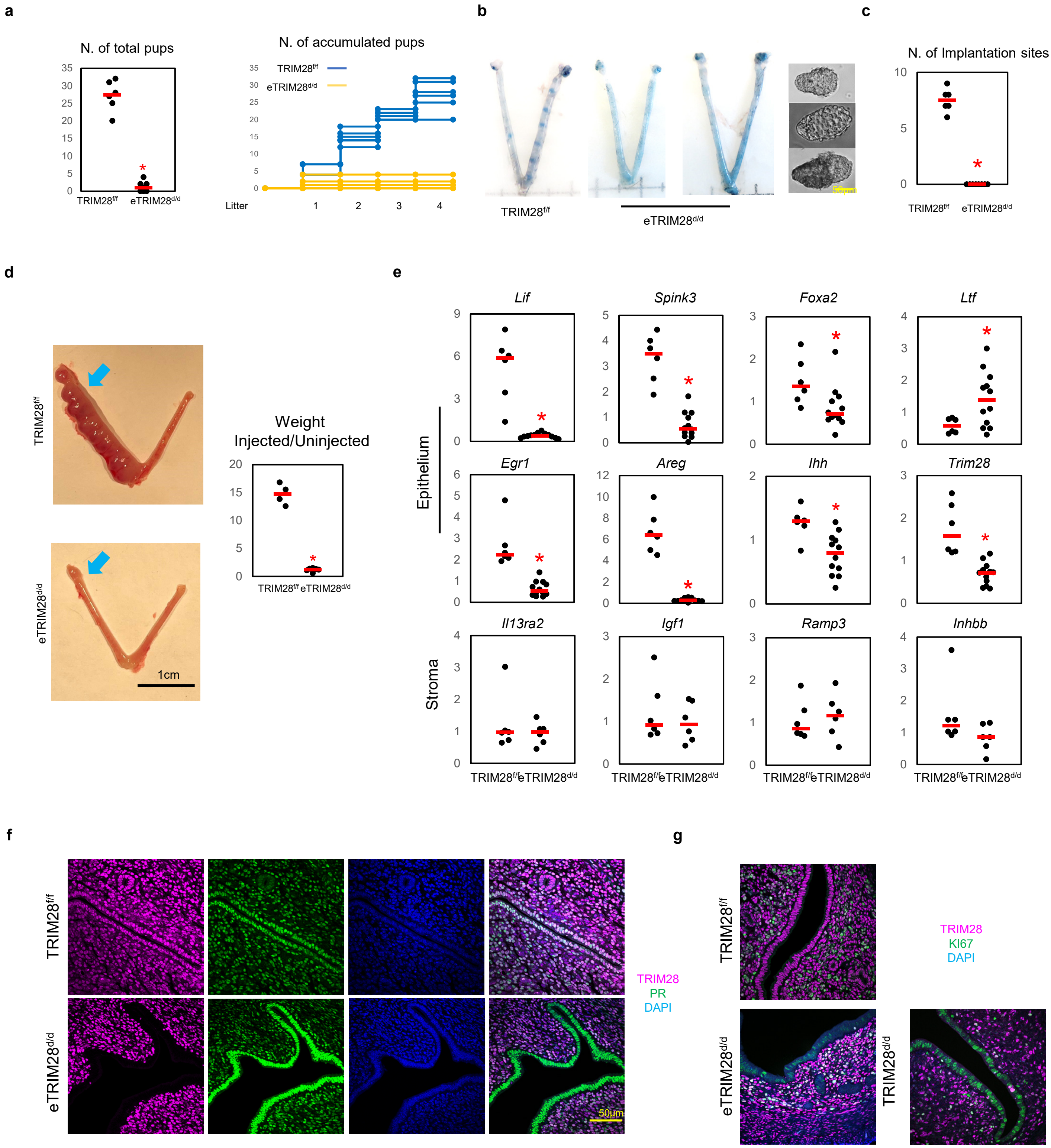

Supplementary Fig. 6. Defective fertility of eTRIM28$^{d/d}$ mice. **a**. The number of total delivered pups during 6-month breeding trial. The line plot of accumulated pups from the 1st to the 4th litter. **b**. The representative uterine pictures at D4.5. Red arrows pointed to the embryo implantation sites. Blastocysts flushed out from the eTRIM28$^{d/d}$ uterus at D4.5. **c**. The number of embryo implantation sites. **d**. The representative images of artificial decidualization. Arrow points to the uterine horn with oil injection. Weight ratio of injected uterine horn compared to the un-injected horn. N=4 biologically independent samples for TRIM28$^{f/f}$ and N=6 biologically independent samples for eTRIM28$^{d/d}$. **e**. The real-time PCR of *Lif*, *Spink3*, *Foxa2*, *Ltf*, *Egr1*, *Areg, Ihh*, *Trim28*, *Il13ra2*, *Igf1*, *Ramp3*, *Inhbb*. N=6 biologically independent samples per group for stromal genes. N=6 biologically independent samples for TRIM28$^{f/f}$ and N=12 biologically independent samples for eTRIM28$^{d/d}$ for epithelial genes. **f**. The immunofluorescence of TRIM28 (red) and PR (green). **g**. The immunofluorescence (IF) of TRIM28 and KI67 in D3.5 TRIM28$^{f/f}$ and eTRIM28$^{d/d}$ uterus. Two-sided student's t test. *p<0.05. The IF was repeated in three different mice per group with similar results.

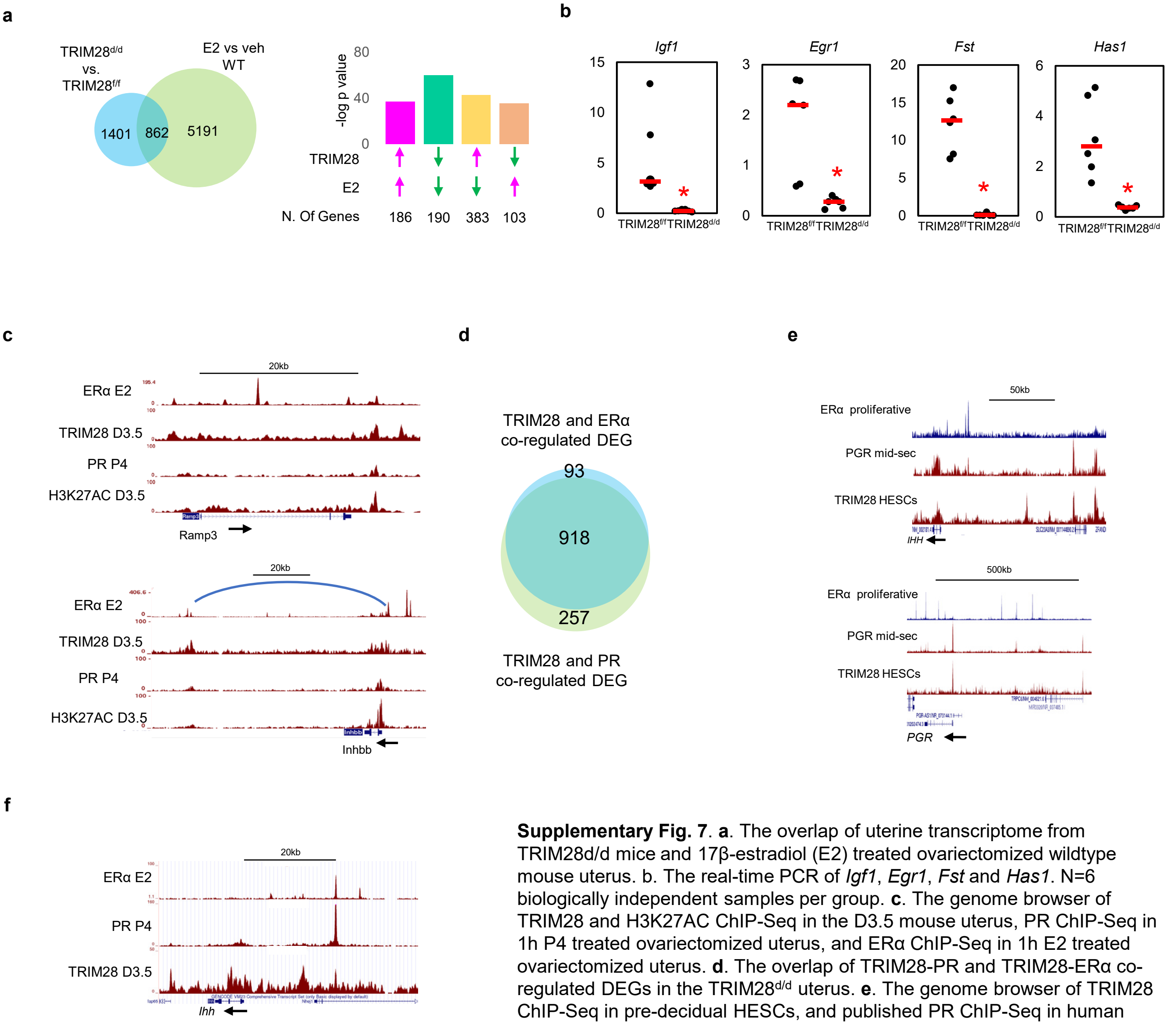

Supplementary Fig. 7. **a**. The overlap of uterine transcriptome from TRIM28d/d mice and 17β-estradiol (E2) treated ovariectomized wildtype mouse uterus. b. The real-time PCR of *Igf1*, *Egr1*, *Fst* and *Has1*. N=6 biologically independent samples per group. **c**. The genome browser of TRIM28 and H3K27AC ChIP-Seq in the D3.5 mouse uterus, PR ChIP-Seq in 1h P4 treated ovariectomized uterus, and ERα ChIP-Seq in 1h E2 treated ovariectomized uterus. **d**. The overlap of TRIM28-PR and TRIM28-ERα co-regulated DEGs in the TRIM28$^{d/d}$ uterus. **e**. The genome browser of TRIM28 ChIP-Seq in pre-decidual HESCs, and published PR ChIP-Seq in human endometrium at mid-secretory phase, ERα ChIP-Seq in human endometrium at proliferative phase. **f**. The genome browser of TRIM28 in the D3.5 mouse uterus, PR ChIP-Seq in 1h P4 treated ovariectomized uterus, and ERα ChIP-Seq in 1h E2 treated ovariectomized uterus at *Ihh* 19kb enhancer. Two-sided student's t test. *p<0.05.

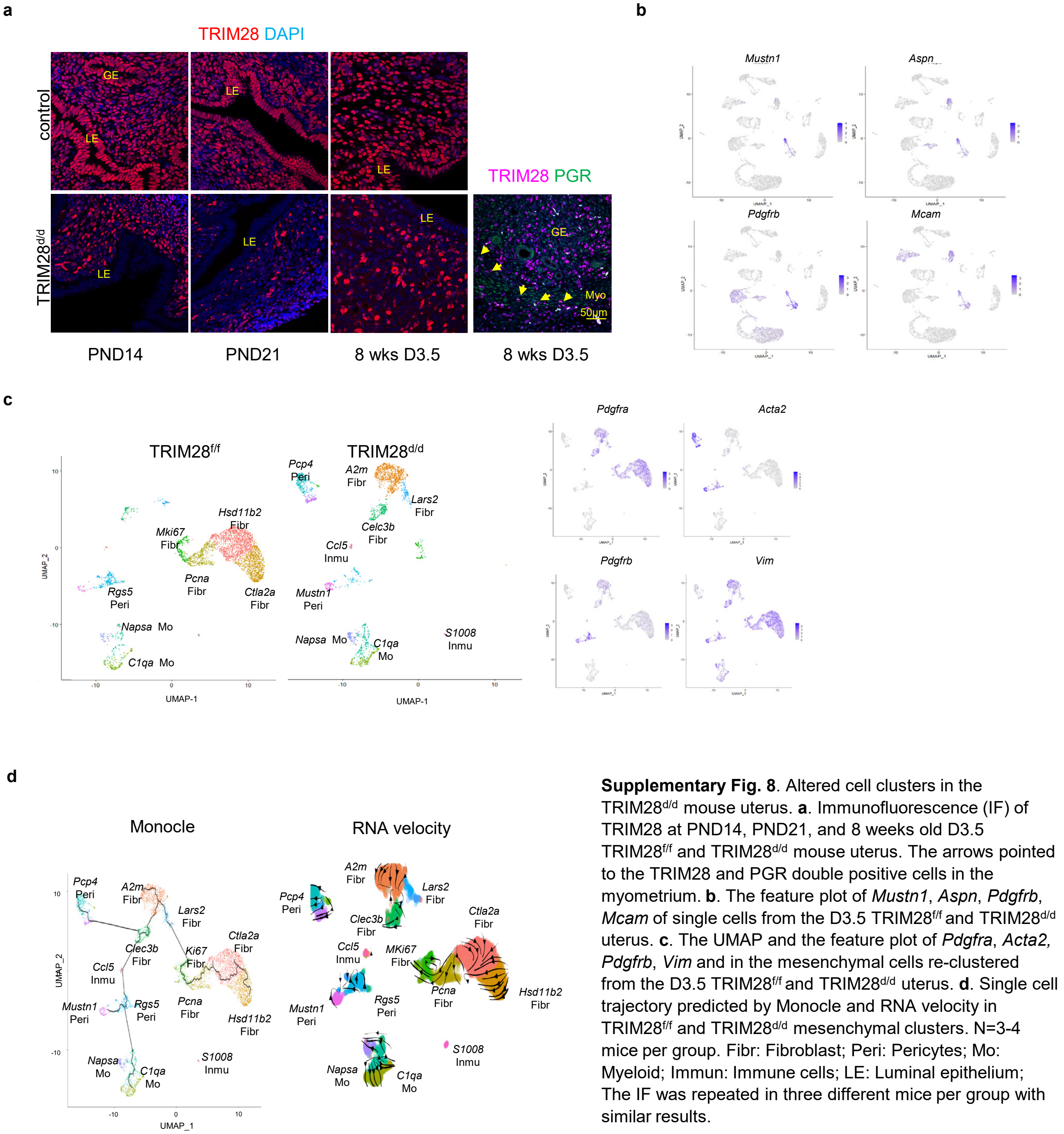

Supplementary Fig. 8. Altered cell clusters in the TRIM28$^{d/d}$ mouse uterus. **a**. Immunofluorescence (IF) of TRIM28 at PND14, PND21, and 8 weeks old D3.5 TRIM28$^{f/f}$ and TRIM28$^{d/d}$ mouse uterus. The arrows pointed to the TRIM28 and PGR double positive cells in the myometrium. **b**. The feature plot of *Mustn1*, *Aspn*, *Pdgfrb*, *Mcam* of single cells from the D3.5 TRIM28$^{f/f}$ and TRIM28$^{d/d}$ uterus. **c**. The UMAP and the feature plot of *Pdgfra*, *Acta2, Pdgfrb*, *Vim* and in the mesenchymal cells re-clustered from the D3.5 TRIM28$^{f/f}$ and TRIM28$^{d/d}$ uterus. **d**. Single cell trajectory predicted by Monocle and RNA velocity in TRIM28$^{f/f}$ and TRIM28$^{d/d}$ mesenchymal clusters. N=3-4 mice per group. Fibr: Fibroblast; Peri: Pericytes; Mo: Myeloid; Immun: Immune cells; LE: Luminal epithelium; The IF was repeated in three different mice per group with similar results.

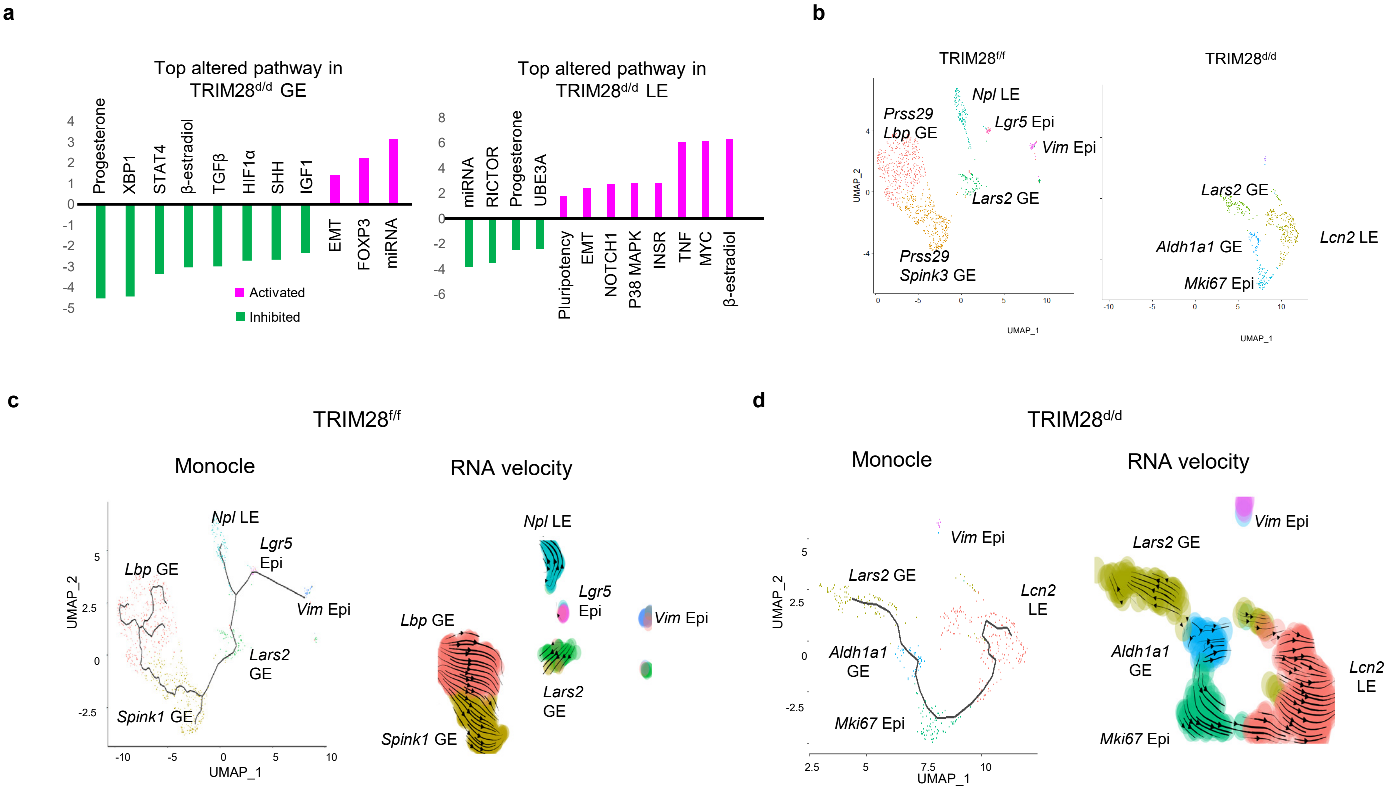

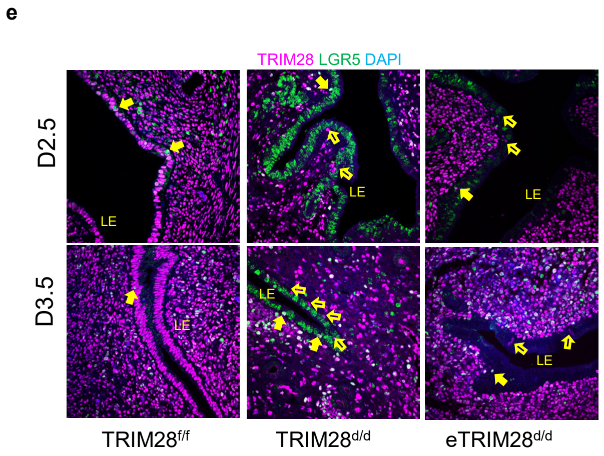

Supplementary Fig. 9. Altered cell clusters in the TRIM28$^{d/d}$ epithelium. **a**. The top altered pathways in the *Lars2* GE from the TRIM28$^{d/d}$ compared to the TRIM28$^{f/f}$ mice. Top altered pathways in the D3.5 *Lcn2* LE from the TRIM28$^{d/d}$ mice compared to *Npl* LE from the TRIM28$^{f/f}$ mice. **b**. The UMAP of all the epithelial cells re-clustered from the D3.5 TRIM28$^{f/f}$ and TRIM28$^{d/d}$ uterus. The cell clusters were labeled with different colors. Single cell trajectory predicted by Monocle and RNA velocity in TRIM28$^{f/f}$ (**c**) and TRIM28$^{d/d}$ (**d**) epithelial clusters. **e**. The immunofluorescence (IF) of TRIM28 and LGR5 at D2.5 and D3.5 in TRIM28$^{f/f}$, TRIM28$^{d/d}$ and eTRIM28$^{d/d}$ uterus. Closed arrow refers to TRIM28 and LGR5 positive epithelium. Open arrow refers to TRIM28 positive but LGR5 negative epithelium. LE: Luminal epithelium; GE: Glandular epithelium; Epi: Epithelium. N=3-4 mice per group. The IF was was repeated in three different mice per group with similar results.
