# Supplementary material for: TRIM28 modulates nuclear receptor signaling to regulate uterine function
Source: Nat Commun. 2023 Aug 1;14:4605. doi: 10.1038/s41467-023-40395-7 (PMC10393996; doi:10.1038/s41467-023-40395-7)
Supplement: Supplementary file 3 — Description of Additional Supplementary Files Document [file 41467_2023_40395_MOESM3_ESM.docx]

**Description of Additional Supplementary Files Document**

**Supplementary Data 1**

Description: One excel file of TRIM28 induced transcriptomic changes in HESCs with three separate sheets.

-The raw data of PR RIME in decidual HESCs. (two PR and two IgG replicates)

-siTRIM28 treatment induced DEGs in decidual HESCs (FC>1.5 or <-1.5, p value< 0.05) and the associated pathway analysis by IPA

- siTRIM28 treatment induced DEGs in pre-decidual HESCs (FC>1.5 or <-1.5, p value< 0.05) and the associated pathway analysis by IPA

**Supplementary Data 2**

Description: One excel file of the integration of TRIM28 RNA-seq, ChIP-Seq and ATA-seq, IP-mass spec withfour separate sheets.

-The DEGs in decidual HESCs that are altered by siPGR and siTRIM28 treatment and closely associated with PR and TRIM28 binding peaks in decidual HESCs; The pathway analysis of these genes using IPA.

- The DEGs in pre-decidual HESCs that are altered by siTRIM28 and closely associated with TRIM28 binding peaks in the pre-decidual HESCs.

- The DEGs in pre-decidual HESCs that are altered by siTRIM28 and closely associated with the chromatin with increased accessibility in the pre-decidual HESCs.

-The raw data of TRIM28 Immunoprecipitation mass spectrometry in pre-decidual HESCs. (one IgG and one TRIM28)

**Supplementary Data 3**

Description: One excel file of TRIM28 induced transcriptomic changes in mouse uterus with four separate sheets.

-The DEGs in mutant TRIM28^d/d^ mouse uterus compared to control (FC>2 or <-2, p value <0.05). The pathway analysis of these genes using IPA.

- The DEGs in control ovariectomized Pgr^cre/+^ mouse uterus 6h after P4 treatment compared to vehicle treatment (FC>2 or <-2, p value <0.05).

-The overlapped DEGs between TRIM28^d/d^ mouse uterus and P4 treated Pgr^cre/+^ mouse uterus (FC>2 or <-2, p value <0.05). The pathway analysis of these genes using IPA.

- The overlapped DEGs between TRIM28^d/d^ mouse uterus and the epithelial DEGs of epithelial PR knockout mice (Ltf^icre/+^PGR^f/f^ vs. PGR^f/f^), the epithelial DEGs of epithelial PR-B overexpression mice (Wnt7a^cre^PGRB^LsL/+^ vs. Wnt7a^cre^).

**Supplementary Data 4**

Description: One excel file of TRIM28 ChIP results in mouse uterus with six separate sheets.

-The overlapped peaks between PR and TRIM28 in mouse uterus.

-The overlapped peaks of PR, TRIM28 and chromatin loop in mouse uterus.

- The overlapped DEGs between TRIM28^d/d^ mouse uterus and the epithelial DEGs of epithelial PR knockout mice (Ltf^icre/+^PGR^f/f^ vs. PGR^f/f^), the epithelial DEGs of epithelial PR-B overexpression mice (Wnt7a^cre^PGRB^LsL/+^ vs. Wnt7a^cre^) which are closely associated with the overlapped PR, TRIM28 and chromatin loops.

-The overlapped peaks between ERα and TRIM28 in mouse uterus.

-The overlapped peaks of ERα, TRIM28 and chromatin loop in mouse uterus.

- The overlapped DEGs between TRIM28^d/d^ mouse uterus and E2 6h treatment in ovariectomized wildtype mouse uterus which are closely associated with the overlapped ERα, TRIM28 and chromatin loops. The pathway analysis of these genes using IPA.

**Supplementary Data 5**

Description: One excel file of scRNA-seq with eight separate sheets.

-The markers genes list of each single cell clusters in the whole uterus.

-The *Pgr*, *Trim28* expression of each single cell clusters in the whole uterus.

-The DEGs and associated pathway analysis between mutant *A2m* Fibr and control *Hsd11b2* Fibr.

-The markers genes list of each single cell clusters in the all the mesenchyme.

-The DEGs and associated pathway analysis between mutant and control *Lars2* GE.

-The DEGs and associated pathway analysis between mutant *Lcn2* LE and control *Npl* LE.

-The markers genes list of each single cell clusters in the all the epithelium.

**Supplementary Data 6**

Description: One excel file of antibody and primers with two separate sheets.

-Antibody catalog number, vendor and dilution

-Primers for RT-PCR and ChIP-PCR

**Supplementary Data 7**

Description: The uncropped gel images of all the western blot in Figure 1, 6 and Supplementary Figure 4.

**Supplementary Data 8**

Description: The individual number for each dotplot in Figure 2, 3, 4, 5, 6, 9 and Supplementary Figure 1, 3, 4, 5, 6.
