# Supplementary material for: TRIM28 modulates nuclear receptor signaling to regulate uterine function
Source: Nat Commun. 2023 Aug 1;14:4605. doi: 10.1038/s41467-023-40395-7 (PMC10393996; doi:10.1038/s41467-023-40395-7)
Supplement: Supplementary file 10 — Supplementary Data 7 [file 41467_2023_40395_MOESM10_ESM.pdf]

Gel Images for Figure 1

TRIM28 antibody: ab22553 abcam

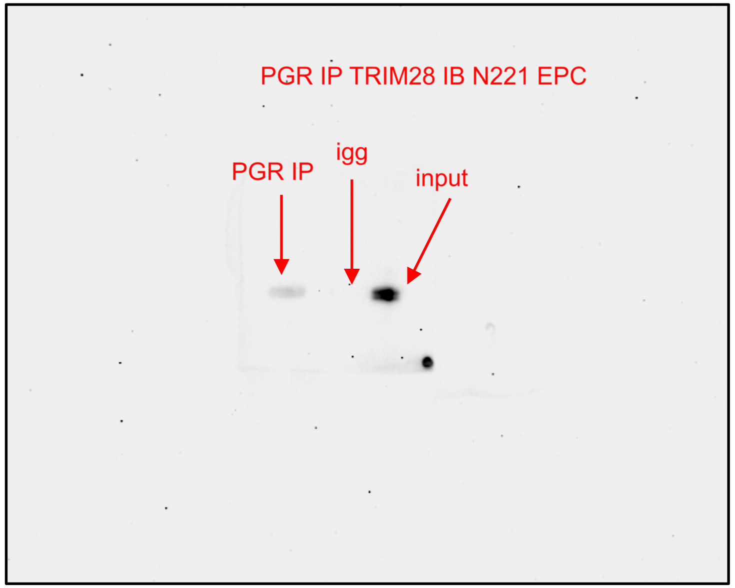

HA antibody: 3724, Cell signaling

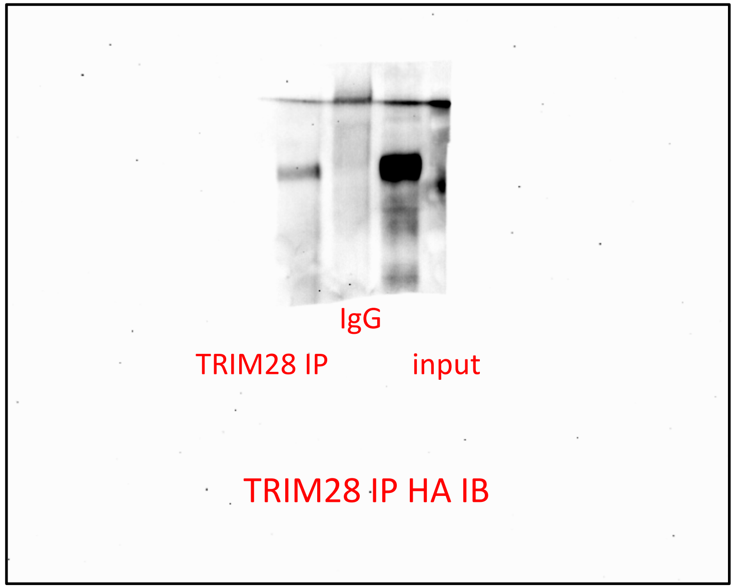

TRIM28 antibody: ab109545 Abcam

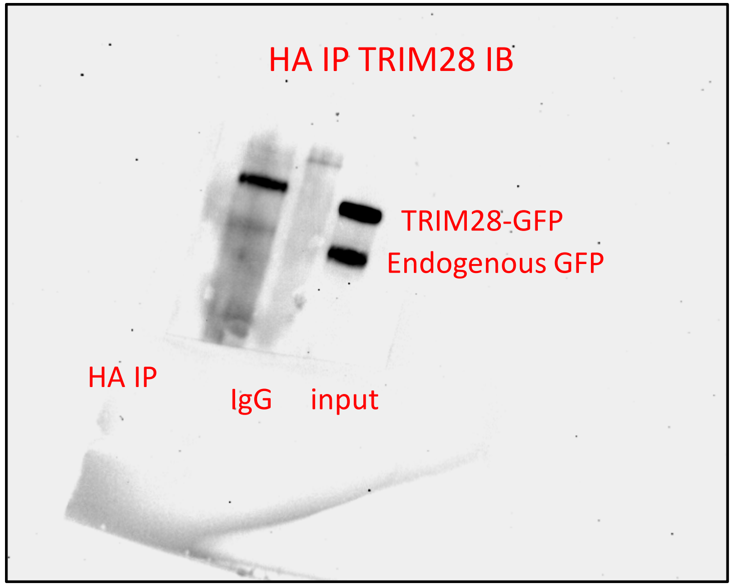

Gel Images for Figure 6

TRIM28 antibody: ab22553 abcam

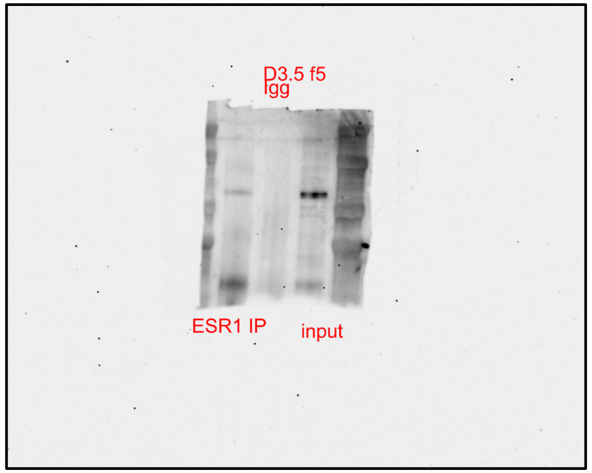

ESR1 antibody: 06-936 Milipore

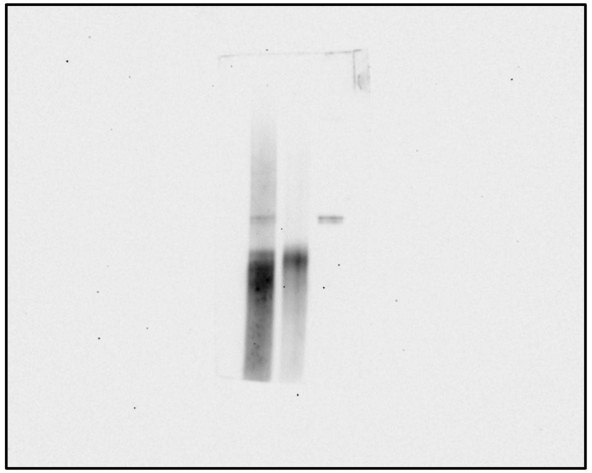

TRIM28 antibody: ab22553 abcam

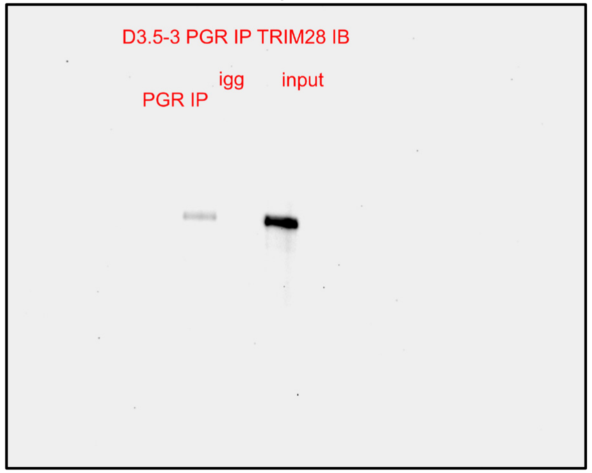

ESR1 antibody: 06-936 Milipore

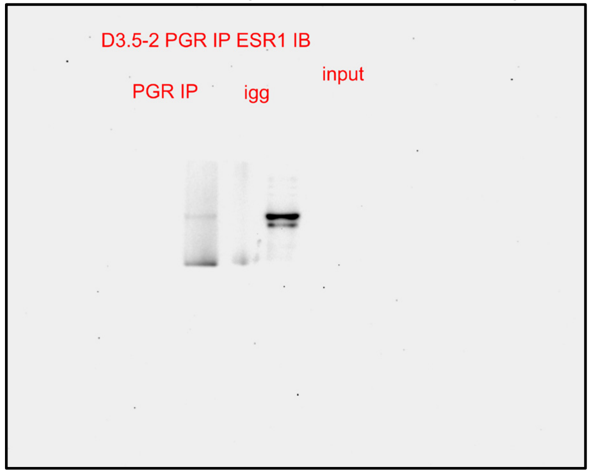

Gel Images for Supplementary Figure 4

TRIM28 antibody: ab109545 Abcam

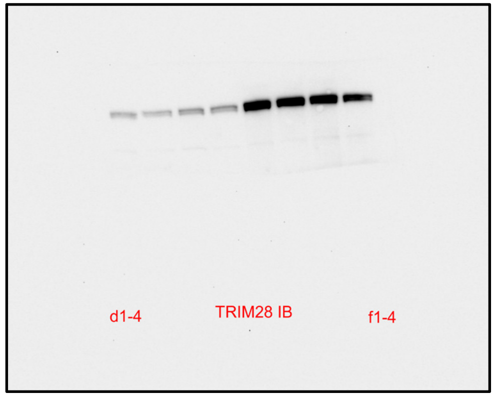

PGR antibody: 1294 Agilent

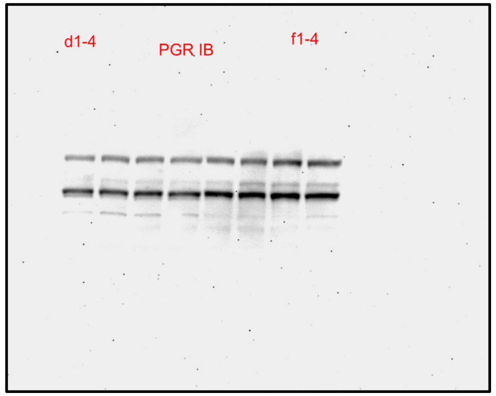

ERα antibody: 06-935 Millipore

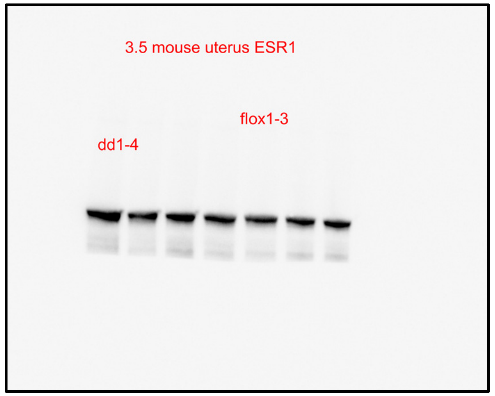

β-actin antibody: A5316 Sigma

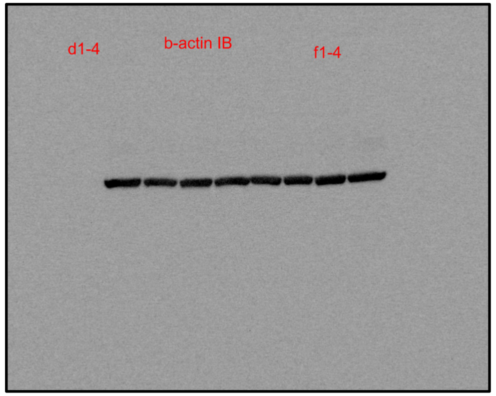

β-actin antibody: A5316 Sigma

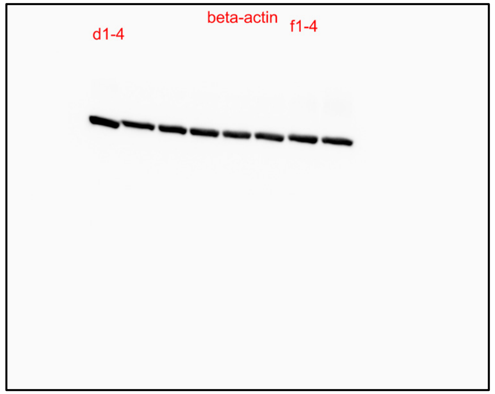

GAPDH antibody: 5174 Cell signaling

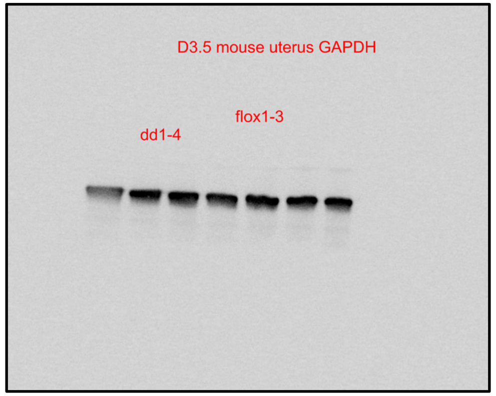

Only one one loading control gel image, GAPDH was shown in the Supplementary figure 4.
