# Supplementary material for: TRIM28 modulates nuclear receptor signaling to regulate uterine function
Source: Nat Commun. 2023 Aug 1;14:4605. doi: 10.1038/s41467-023-40395-7 (PMC10393996; doi:10.1038/s41467-023-40395-7)
Supplement: Supplementary file 12 — Reporting Summary [file 41467_2023_40395_MOESM12_ESM.pdf]

nature portfolio

Corresponding author(s): *Francesco J DeMayo*

Last updated by author(s): Jul 3, 2023

Reporting Summary

Nature Portfolio wishes to improve the reproducibility of the work that we publish. This form provides structure for consistency and transparency in reporting. For further information on Nature Portfolio policies, see our Editorial Policies and the Editorial Policy Checklist.

Statistics

For all statistical analyses, confirm that the following items are present in the figure legend, table legend, main text, or Methods section.

n/a	Confirmed	
☐	☒	The exact sample size (*n*) for each experimental group/condition, given as a discrete number and unit of measurement
☐	☒	A statement on whether measurements were taken from distinct samples or whether the same sample was measured repeatedly
☐	☒	The statistical test(s) used AND whether they are one- or two-sided<br>*Only common tests should be described solely by name; describe more complex techniques in the Methods section.*
☒	☐	A description of all covariates tested
☐	☒	A description of any assumptions or corrections, such as tests of normality and adjustment for multiple comparisons
☐	☒	A full description of the statistical parameters including central tendency (e.g. means) or other basic estimates (e.g. regression coefficient) AND variation (e.g. standard deviation) or associated estimates of uncertainty (e.g. confidence intervals)
☐	☒	For null hypothesis testing, the test statistic (e.g. *F*, *t*, *r*) with confidence intervals, effect sizes, degrees of freedom and *P* value noted<br>*Give P values as exact values whenever suitable.*
☒	☐	For Bayesian analysis, information on the choice of priors and Markov chain Monte Carlo settings
☒	☐	For hierarchical and complex designs, identification of the appropriate level for tests and full reporting of outcomes
☒	☐	Estimates of effect sizes (e.g. Cohen's *d*, Pearson's *r*), indicating how they were calculated

Our web collection on statistics for biologists contains articles on many of the points above.

Software and code

Policy information about availability of computer code

Data collection	No software used for data collections
Data analysis	*Tophat (v2.0.4), Cufflinks (v2.0.2), bedGraphToBigWig, EdgeR, SEMIRP R package, Partek Genomics Suite 6.6, Nextbio, Picard-tools-1.96, MACS2, trim_galore (v0.4.4), EaSeq, GREAT, Homer, Zeiss black edition, Spectrum Mill software package , PEAKS Studio version 8 built 20.*

For manuscripts utilizing custom algorithms or software that are central to the research but not yet described in published literature, software must be made available to editors and reviewers. We strongly encourage code deposition in a community repository (e.g. GitHub). See the Nature Portfolio guidelines for submitting code & software for further information.

Data

Policy information about availability of data

All manuscripts must include a data availability statement. This statement should provide the following information, where applicable:

- Accession codes, unique identifiers, or web links for publicly available datasets
- A description of any restrictions on data availability
- For clinical datasets or third party data, please ensure that the statement adheres to our policy

All the raw and processed data of RNA-Seq, ATAC-Seq, ChIP-Seq and scRNA-Seq were uploaded to GSE205481.

March 2021

Human research participants

Policy information about studies involving human research participants and Sex and Gender in Research.

Reporting on sex and gender	*No human participants were included in this study.*
Population characteristics	*Describe the covariate-relevant population characteristics of the human research participants (e.g. age, genotypic information, past and current diagnosis and treatment categories). If you filled out the behavioural & social sciences study design questions and have nothing to add here, write "See above."*
Recruitment	*Describe how participants were recruited. Outline any potential self-selection bias or other biases that may be present and how these are likely to impact results.*
Ethics oversight	*Identify the organization(s) that approved the study protocol.*

Note that full information on the approval of the study protocol must also be provided in the manuscript.

Field-specific reporting

Please select the one below that is the best fit for your research. If you are not sure, read the appropriate sections before making your selection.

☒ Life sciences ☐ Behavioural & social sciences ☐ Ecological, evolutionary & environmental sciences

For a reference copy of the document with all sections, see nature.com/documents/nr-reporting-summary-flat.pdf

Life sciences study design

All studies must disclose on these points even when the disclosure is negative.

Sample size	*Based on our previous work, a sample size of 6 mice will provide 80% statistical power (beta=0.8) if there is a ≥ 20% difference between experimental groups when alpha is p<0.05. Therefore, we used at lease 6 different mice for each group were included for all the mouse experiments. For the RNA-seq of human endometrial stromal cells with different treatment, three primary cell lines from different donors were included for the analysis. For CUT&RUN, ChIP-seq, ATAC-seq,analysis, we repeated either in two different mice or two primary cell lines from different donor as the duplicate samples. The scRNA-seq analysis pooled three control mice as one control sample and pooled four mutant mice as one mutant sample. The RIME was conducted on the pooled primary cell lines from three different donor with two technical replicates.*
Data exclusions	*No data excluded.*
Replication	*At least six biological replicates were used for each group in the student's t test. Two biological replicates were used for RNA-seq, ChIP-seq, and ATAC-seq. One replicate from three or four mice from the same group was used for scRNA-seq. Two technical replicates from pooled primary cell lines of three different donors were used for RIME*
Randomization	*The primary cells were cultured, mixed, counted, and randomly seeded into the 6-well plate with either siNT or siTRIM28 treatment. For the mouse study, we will randomly allocate the mutant mice and their control littermates into the same experiments.*
Blinding	The investigator was not blinded to the data during collection and analysis since the same investigator did all this.

Behavioural & social sciences study design

All studies must disclose on these points even when the disclosure is negative.

Study description	*Briefly describe the study type including whether data are quantitative, qualitative, or mixed-methods (e.g. qualitative cross-sectional, quantitative experimental, mixed-methods case study).*
Research sample	*State the research sample (e.g. Harvard university undergraduates, villagers in rural India) and provide relevant demographic information (e.g. age, sex) and indicate whether the sample is representative. Provide a rationale for the study sample chosen. For studies involving existing datasets, please describe the dataset and source.*
Sampling strategy	*Describe the sampling procedure (e.g. random, snowball, stratified, convenience). Describe the statistical methods that were used to predetermine sample size OR if no sample-size calculation was performed, describe how sample sizes were chosen and provide a rationale for why these sample sizes are sufficient. For qualitative data, please indicate whether data saturation was considered, and what criteria were used to decide that no further sampling was needed.*
Data collection	*Provide details about the data collection procedure, including the instruments or devices used to record the data (e.g. pen and paper,*

Data collection	*computer, eye tracker, video or audio equipment) whether anyone was present besides the participant(s) and the researcher, and whether the researcher was blind to experimental condition and/or the study hypothesis during data collection.*
Timing	*Indicate the start and stop dates of data collection. If there is a gap between collection periods, state the dates for each sample cohort.*
Data exclusions	*If no data were excluded from the analyses, state so OR if data were excluded, provide the exact number of exclusions and the rationale behind them, indicating whether exclusion criteria were pre-established.*
Non-participation	*State how many participants dropped out/declined participation and the reason(s) given OR provide response rate OR state that no participants dropped out/declined participation.*
Randomization	*If participants were not allocated into experimental groups, state so OR describe how participants were allocated to groups, and if allocation was not random, describe how covariates were controlled.*

Ecological, evolutionary & environmental sciences study design

All studies must disclose on these points even when the disclosure is negative.

Study description	*Briefly describe the study. For quantitative data include treatment factors and interactions, design structure (e.g. factorial, nested, hierarchical), nature and number of experimental units and replicates.*
Research sample	*Describe the research sample (e.g. a group of tagged Passer domesticus, all Stenocereus thurberi within Organ Pipe Cactus National Monument), and provide a rationale for the sample choice. When relevant, describe the organism taxa, source, sex, age range and any manipulations. State what population the sample is meant to represent when applicable. For studies involving existing datasets, describe the data and its source.*
Sampling strategy	*Note the sampling procedure. Describe the statistical methods that were used to predetermine sample size OR if no sample-size calculation was performed, describe how sample sizes were chosen and provide a rationale for why these sample sizes are sufficient.*
Data collection	*Describe the data collection procedure, including who recorded the data and how.*
Timing and spatial scale	*Indicate the start and stop dates of data collection, noting the frequency and periodicity of sampling and providing a rationale for these choices. If there is a gap between collection periods, state the dates for each sample cohort. Specify the spatial scale from which the data are taken*
Data exclusions	*If no data were excluded from the analyses, state so OR if data were excluded, describe the exclusions and the rationale behind them, indicating whether exclusion criteria were pre-established.*
Reproducibility	*Describe the measures taken to verify the reproducibility of experimental findings. For each experiment, note whether any attempts to repeat the experiment failed OR state that all attempts to repeat the experiment were successful.*
Randomization	*Describe how samples/organisms/participants were allocated into groups. If allocation was not random, describe how covariates were controlled. If this is not relevant to your study, explain why.*
Blinding	*Describe the extent of blinding used during data acquisition and analysis. If blinding was not possible, describe why OR explain why blinding was not relevant to your study.*

Did the study involve field work? ☐ Yes ☐ No

Field work, collection and transport

Field conditions	*Describe the study conditions for field work, providing relevant parameters (e.g. temperature, rainfall).*
Location	*State the location of the sampling or experiment, providing relevant parameters (e.g. latitude and longitude, elevation, water depth).*
Access & import/export	*Describe the efforts you have made to access habitats and to collect and import/export your samples in a responsible manner and in compliance with local, national and international laws, noting any permits that were obtained (give the name of the issuing authority, the date of issue, and any identifying information).*
Disturbance	*Describe any disturbance caused by the study and how it was minimized.*

Reporting for specific materials, systems and methods

We require information from authors about some types of materials, experimental systems and methods used in many studies. Here, indicate whether each material, system or method listed is relevant to your study. If you are not sure if a list item applies to your research, read the appropriate section before selecting a response.

Materials & experimental systems

n/a	Involved in the study
☐	☒ Antibodies
☐	☒ Eukaryotic cell lines
☐	☐ Palaeontology and archaeology
☐	☒ Animals and other organisms
☐	☐ Clinical data
☐	☐ Dual use research of concern

Methods

n/a	Involved in the study
☐	☒ ChIP-seq
☐	☐ Flow cytometry
☐	☐ MRI-based neuroimaging

Antibodies

Antibodies used

PR (sc7208, Santa Cruz); Rabbit IgG (2729, Cell signaling); PR (8757, Cell signaling); PR (1294, Agilent); H3K27AC (39133, Active motif); H3K27me3 (39155, Active motif); HA-tag (3724, Cell signaling); TRIM28 (ab10484, Abcam); TRIM28 (ab109545, Abcam); TRIM28 (ab22553, Abcam); ERα (1D5, Biocare medical); LGR5 (LS-A1232-50, LS-Bio); FOXO1 (2880, Cell signaling); GJB2 (33-5800, Invitrogen); COX2 (12282, Cell signaling); GFP (ab6673, Abcam); MUC1 (ab109185, Abcam); FOXA2 (8186, Abcam); VIM (sc7557, Santa Cruz); αSMA (ab5694, Abcam); KI67 (ab15580, Abcam); CD31 (ab28364, Abcam); HAND2 (sc9409, Santa Cruz); ERα (06-935, Millipore); GAPDH (5174, Cell signaling); ERα (sc542, Santa Cruz).

Validation

PR (sc7208, Santa Cruz) has been cited for PR ChIP-seq in both human and mouse (sc542, Santa Cruz) has been cited for ChIPseq in "Chi, R. A. et al. Human Endometrial Transcriptome and Progesterone Receptor Cistrome Reveal Important Pathways and Epithelial Regulators. J Clin Endocrinol Metab 105 (2020)" https://doi.org:10.1210/clinem/dgz117" and "Rubel, C. A. et al. Research Resource: Genome-Wide Profiling of Progesterone Receptor Binding in the Mouse Uterus. Molecular Endocrinology 26, 1428-1442 (2012). https://doi.org:10.1210/me.2011-1355". ERα (sc542, Santa Cruz) has been cited for ER ChIP-seq in mouse "Hewitt, S. C. et al. Research resource: whole-genome estrogen receptor alpha binding in mouse uterine tissue revealed by ChIP-seq. Mol Endocrinol 26, 887-898 (2012). https://doi.org:10.1210/me.2011-1311". H3K27AC (39133, Active motif) has been validated for ChIP by vendor https://www.activemotif.com/catalog/details/39133/histone-h3-acetyl-lys27-antibody-pab. H3K27me3 (39155, Active motif) has been validated for ChIP by vendor https://www.activemotif.com/catalog/details/39155/histone-h3-trimethyl-lys27-antibody-pab. Rabbit IgG (2729, Cell signaling) was validated for IP and ChIP by the vendor https://www.cellsignal.com/products/primary-antibodies/normal-rabbit-igg/2729. PR (8757, Cell signaling) was validated for IF, IP, ChIP in human by the vendor https://www.cellsignal.com/products/primary-antibodies/progesterone-receptor-a-b-d8q2j-xp-rabbit-mab/8757, and cited for IHC in mouse by "Li, R. et al. The role of epithelial progesterone receptor isoforms in embryo implantation. iScience 24, 103487 (2021). https://doi.org:10.1016/j.isci.2021.103487". PR (1294, Agilent) has been validated for western by vendor https://www.agilent.com/en/product/immunohistochemistry/antibodies-controls/primary-antibodies/progesterone-receptor-%28concentrate%29-76578#specifications. HA-tag (3724, Cell signaling) has been validated for IP and western by the vendor https://www.cellsignal.com/products/primary-antibodies/ha-tag-c29f4-rabbit-mab/3724. TRIM28 (ab10484, Abcam) has been validated by vendor for IHC, IP, IF in human and mouse https://www.abcam.com/kap1-antibody-ab10484.html and cited for ChIP in mouse by "He, H., Ye, A., Kim, H. & Kim, J. PEG3 Interacts with KAP1 through KRAB-A. Plos One 11 (2016). https://doi.org:ARTN e016754110.1371/journal.pone.0167541" and in human by "Bunch, H. et al. TRIM28 regulates RNA polymerase II promoter-proximal pausing and pause release. Nat Struct Mol Biol 21, 876-883 (2014). https://doi.org:10.1038/nsmb.2878". TRIM28 (ab109545, Abcam) has been validated for IHC, IF in human by vendor https://www.abcam.com/kap1-antibody-epr5249-ab109545.html. TRIM28 (ab22553, Abcam) has been validated for WB, IHC in human by vendor https://www.abcam.com/kap1-antibody-20c1-ab22553.html. ERα (1D5, Biocare medical), αSMA (ab5694, Abcam), FOXA2 (8186, Abcam), KI67 (ab15580, Abcam); has been cited for IHC in mouse by "Li, R. et al. Increased FOXL2 expression alters uterine structures and functionsdagger. Biol Reprod 103, 951-965 (2020). https://doi.org:10.1093/biolre/ioaa143". LGR5 (LS-A1232-50, LS-Bio) has been validated for IHC in human by vendor https://www.lsbio.com/pathplus-antibodies/pathplus-gpr49-antibody-lgr5-antibody-n-terminus-ihc-ls-a1232/187427. FOXO1 (2880, Cell signaling); GJB2 (33-5800, Invitrogen); MUC1 (ab109185, Abcam) has been cited for IHC in mouse by "Vasquez, Y. M. et al. FOXO1 regulates uterine epithelial integrity and progesterone receptor expression critical for embryo implantation. PLoS Genet 14, e1007787 (2018). https://doi.org:10.1371/journal.pgen.1007787". COX2 (12282, Cell signaling) has been validated for IHC in mouse by vendor https://www.cellsignal.com/products/primary-antibodies/cox2-d5h5-xp-rabbit-mab/12282. GFP (ab6673, Abcam) has been validated for IF by vendor https://www.abcam.com/gfp-antibody-ab6673.html. VIM (sc7557, Santa Cruz) has been cited for IF in mouse by "Alves, S. et al. Lentiviral vector-mediated overexpression of mutant ataxin-7 recapitulates SCA7 pathology and promotes accumulation of the FUS/TLS and MBNL1 RNA-binding proteins. Mol Neurodegener 11, 58 (2016). https://doi.org:10.1186/s13024-016-0123-2". CD31 (ab28364, Abcam) has been predicted to be working for IHC in mouse by vendor https://www.abcam.com/cd31-antibody-ab28364.html. HAND2 (sc9409, Santa Cruz) has been cited for IHC In mouse by "Cooke, P. S. et al. Brief exposure to progesterone during a critical neonatal window prevents uterine gland formation in mice. Biol Reprod 86, 63 (2012). https://doi.org:10.1095/biolreprod.111.097188". GAPDH (5174, Cell signaling) has been validated for WB in human and mouse by vendor https://www.cellsignal.com/products/primary-antibodies/gapdh-d16h11-xp-rabbit-mab/5174. ERα (06-935, Millipore) has been validated for IP and western in human by vendor https://www.emdmillipore.com/US/en/product/Anti-Estrogen-Receptor-Antibody,MM_NF-06-935#.

Eukaryotic cell lines

Policy information about cell lines and Sex and Gender in Research

Cell line source(s)

The three primary human endometrial cell samples were collected from different female donors.

Authentication	No
Mycoplasma contamination	Not tested.
Commonly misidentified lines (See ICLAC register)	*Name any commonly misidentified cell lines used in the study and provide a rationale for their use.*

Palaeontology and Archaeology

Specimen provenance	*Provide provenance information for specimens and describe permits that were obtained for the work (including the name of the issuing authority, the date of issue, and any identifying information). Permits should encompass collection and, where applicable, export.*
Specimen deposition	*Indicate where the specimens have been deposited to permit free access by other researchers.*
Dating methods	*If new dates are provided, describe how they were obtained (e.g. collection, storage, sample pretreatment and measurement), where they were obtained (i.e. lab name), the calibration program and the protocol for quality assurance OR state that no new dates are provided.*

☐ Tick this box to confirm that the raw and calibrated dates are available in the paper or in Supplementary Information.

Ethics oversight	*Identify the organization(s) that approved or provided guidance on the study protocol, OR state that no ethical approval or guidance was required and explain why not.*

Note that full information on the approval of the study protocol must also be provided in the manuscript.

Animals and other research organisms

Policy information about studies involving animals; ARRIVE guidelines recommended for reporting animal research, and Sex and Gender in Research

Laboratory animals	All the control or mutant mice were C57BL6/J and all the experiments were initiated at 8 weeks old.
Wild animals	*Provide details on animals observed in or captured in the field; report species and age where possible. Describe how animals were caught and transported and what happened to captive animals after the study (if killed, explain why and describe method; if released, say where and when) OR state that the study did not involve wild animals.*
Reporting on sex	Since we focused on uterine functions, only female mice are included.
Field-collected samples	*For laboratory work with field-collected samples, describe all relevant parameters such as housing, maintenance, temperature, photoperiod and end-of-experiment protocol OR state that the study did not involve samples collected from the field.*
Ethics oversight	All animal studies were conducted in accordance with the Guide for the Care and Use of Laboratory Animals published by the National Institutes of Health and animal protocols approved by the Institutional Animal Care and Use Committee (IACUC) at the National institute of Environmental Health Sciences (NIEHS).

Note that full information on the approval of the study protocol must also be provided in the manuscript.

Clinical data

Policy information about clinical studies

All manuscripts should comply with the ICMJE guidelines for publication of clinical research and a completed CONSORT checklist must be included with all submissions.

Clinical trial registration	*Provide the trial registration number from ClinicalTrials.gov or an equivalent agency.*
Study protocol	*Note where the full trial protocol can be accessed OR if not available, explain why.*
Data collection	*Describe the settings and locales of data collection, noting the time periods of recruitment and data collection.*
Outcomes	*Describe how you pre-defined primary and secondary outcome measures and how you assessed these measures.*

Dual use research of concern

Policy information about dual use research of concern

Hazards

Could the accidental, deliberate or reckless misuse of agents or technologies generated in the work, or the application of information presented in the manuscript, pose a threat to:

No	Yes	
☒	☐	Public health
☒	☐	National security
☒	☐	Crops and/or livestock
☒	☐	Ecosystems
☒	☐	Any other significant area

Experiments of concern

Does the work involve any of these experiments of concern:

No	Yes	
☒	☐	Demonstrate how to render a vaccine ineffective
☒	☐	Confer resistance to therapeutically useful antibiotics or antiviral agents
☒	☐	Enhance the virulence of a pathogen or render a nonpathogen virulent
☒	☐	Increase transmissibility of a pathogen
☒	☐	Alter the host range of a pathogen
☒	☐	Enable evasion of diagnostic/detection modalities
☒	☐	Enable the weaponization of a biological agent or toxin
☒	☐	Any other potentially harmful combination of experiments and agents

ChIP-seq

Data deposition

☒ Confirm that both raw and final processed data have been deposited in a public database such as GEO.

☒ Confirm that you have deposited or provided access to graph files (e.g. BED files) for the called peaks.

Data access links<br>*May remain private before publication.*	To review GEO accession GSE205481:<br>Go to https://www.ncbi.nlm.nih.gov/geo/query/acc.cgi?acc=GSE205481<br>Enter token ivgdiowwhzojdsx into the box
Files in database submission	GSM6213349 P1-Pre-dec-TRIM28<br>GSM6213350 P2-Pre-dec-TRIM28<br>GSM6213351 P1-Pre-dec-input<br>GSM6213352 P2-Pre-dec-input<br>GSM6213353 P1-Pre-dec-H3K27AC<br>GSM6213354 P2-Pre-dec-H3K27AC<br>GSM6213355 P1-Pre-dec-H3K27me3<br>GSM6213356 P2-Pre-dec-H3K27me4<br>GSM6213357 D3.5-TRIM28-ChIP-seq<br>GSM7118780 D3.5-TRIM28-ChIP-seq R2<br>GSM6213358 D3.5-TRIM28-input<br>GSM7080469 D3.5-PGR-ChIP-seq<br>GSM7118779 D3.5-PGR-ChIP-seq R2
Genome browser session<br>(e.g. UCSC)	http://genome.ucsc.edu/cgi-bin/hgTracks?db=mm10&hubUrl=https://orio.niehs.nih.gov/ucscview/DeMayo/Rong/TRIM28-mouse/hub.txt

Methodology

Replicates	TRIM28 and PGR ChIP-seq were performed in the uterus from two D3.5 wildtype mice as biological duplicates.<br>TRIM28, H3K27AC, H3K27me3 were chipped at two primary human endometrial stromal cells from different donors as biological duplicate
Sequencing depth	All the ChIP-seq files are 50bp, single-end. D3.5-PGR-ChIP-seq sequencing depth 122,482,842 with aligned reads 99,669,729. D3.5-PGR-ChIP-seq R2 sequencing depth 82,449,396 with aligned reads 63,670,612. D3.5-TRIM28-ChIP-seq sequencing depth 141,410,881, aligned reads 111,194,874. D3.5-TRIM28-ChIP-seq R2 sequencing depth 74,964,107, aligned reads 54,828,175. D3.5-input sequencing depth 34,605,902, aligned reads 25,412,519; P1-Pre-dec-TRIM28 sequencing depth 89,928,992, aligned reads 60,241,488; P2-Pre-dec-TRIM28 sequencing depth 89,081,387, aligned reads 63,049,504; P1-Pre-dec-input sequencing depth 86485183, aligned reads 65,6114,93; P2-Pre-dec-input sequencing depth 66,927,940, aligned reads 51,434,579; P1-Pre-dec-H3K27AC sequencing depth 69,376,280, aligned reads 56,126,517; P2-Pre-dec-H3K27AC sequencing depth 94,323,730, aligned reads 76,074,918; P1-Pre-dec-H3K27me3 sequencing depth 90,174,629, aligned reads 71,416,012; P2-Pre-dec-H3K27me3 sequencing depth 73,195,614, aligned reads 56,988,830.
Antibodies	PR (sc7208, Santa Cruz); TRIM28 (ab10484, Abcam); H3K27AC (39133, Active motif); H3K27me3 (39155, Active motif).

Peak calling parameters	After trimming the adapter reads and filtering the low-quality reads (average quality scores < 20), the ChIP-seq data were mapped to hg38 or mm10. The duplicate reads were removed using the MarkDuplicates tool in picard-tools-1.96. The retained read alignments were sorted by coordinates, extended to 300 bases, and peaks were called using MACS2 with FDR cutoff at 0.0001.
Data quality	The low-quality reads (average quality scores < 20) were filtered. The duplicate reads were removed. Only FDR less than 0.0001 were called as peaks.
Software	Picard-tools-1.96, MACS2, Homer, EaSeq, GREAT

Flow Cytometry

Plots

Confirm that:

☐ The axis labels state the marker and fluorochrome used (e.g. CD4-FITC).

☐ The axis scales are clearly visible. Include numbers along axes only for bottom left plot of group (a 'group' is an analysis of identical markers).

☐ All plots are contour plots with outliers or pseudocolor plots.

☐ A numerical value for number of cells or percentage (with statistics) is provided.

Methodology

Sample preparation	*Describe the sample preparation, detailing the biological source of the cells and any tissue processing steps used.*
Instrument	*Identify the instrument used for data collection, specifying make and model number.*
Software	*Describe the software used to collect and analyze the flow cytometry data. For custom code that has been deposited into a community repository, provide accession details.*
Cell population abundance	*Describe the abundance of the relevant cell populations within post-sort fractions, providing details on the purity of the samples and how it was determined.*
Gating strategy	*Describe the gating strategy used for all relevant experiments, specifying the preliminary FSC/SSC gates of the starting cell population, indicating where boundaries between "positive" and "negative" staining cell populations are defined.*

☐ Tick this box to confirm that a figure exemplifying the gating strategy is provided in the Supplementary Information.

Magnetic resonance imaging

Experimental design

Design type	*Indicate task or resting state; event-related or block design.*
Design specifications	*Specify the number of blocks, trials or experimental units per session and/or subject, and specify the length of each trial or block (if trials are blocked) and interval between trials.*
Behavioral performance measures	*State number and/or type of variables recorded (e.g. correct button press, response time) and what statistics were used to establish that the subjects were performing the task as expected (e.g. mean, range, and/or standard deviation across subjects).*

Acquisition

Imaging type(s)	*Specify: functional, structural, diffusion, perfusion.*
Field strength	*Specify in Tesla*
Sequence & imaging parameters	*Specify the pulse sequence type (gradient echo, spin echo, etc.), imaging type (EPI, spiral, etc.), field of view, matrix size, slice thickness, orientation and TE/TR/flip angle.*
Area of acquisition	*State whether a whole brain scan was used OR define the area of acquisition, describing how the region was determined.*

Diffusion MRI ☐ Used ☐ Not used

Preprocessing

Preprocessing software	*Provide detail on software version and revision number and on specific parameters (model/functions, brain extraction, segmentation, smoothing kernel size, etc.).*

Normalization | *If data were normalized/standardized, describe the approach(es): specify linear or non-linear and define image types used for transformation OR indicate that data were not normalized and explain rationale for lack of normalization.*

Normalization template | *Describe the template used for normalization/transformation, specifying subject space or group standardized space (e.g. original Talairach, MNI305, ICBM152) OR indicate that the data were not normalized.*

Noise and artifact removal | *Describe your procedure(s) for artifact and structured noise removal, specifying motion parameters, tissue signals and physiological signals (heart rate, respiration).*

Volume censoring | *Define your software and/or method and criteria for volume censoring, and state the extent of such censoring.*

Statistical modeling & inference

Model type and settings | *Specify type (mass univariate, multivariate, RSA, predictive, etc.) and describe essential details of the model at the first and second levels (e.g. fixed, random or mixed effects; drift or auto-correlation).*

Effect(s) tested | *Define precise effect in terms of the task or stimulus conditions instead of psychological concepts and indicate whether ANOVA or factorial designs were used.*

Specify type of analysis: ☐ Whole brain ☐ ROI-based ☐ Both

Statistic type for inference
(See Eklund et al. 2016) | *Specify voxel-wise or cluster-wise and report all relevant parameters for cluster-wise methods.*

Correction | *Describe the type of correction and how it is obtained for multiple comparisons (e.g. FWE, FDR, permutation or Monte Carlo).*

Models & analysis

n/a	Involved in the study
☐	☐ Functional and/or effective connectivity
☐	☐ Graph analysis
☐	☐ Multivariate modeling or predictive analysis

Functional and/or effective connectivity | *Report the measures of dependence used and the model details (e.g. Pearson correlation, partial correlation, mutual information).*

Graph analysis | *Report the dependent variable and connectivity measure, specifying weighted graph or binarized graph, subject- or group-level, and the global and/or node summaries used (e.g. clustering coefficient, efficiency, etc.).*

Multivariate modeling and predictive analysis | *Specify independent variables, features extraction and dimension reduction, model, training and evaluation metrics.*
